# Decoding Schizophrenia: How AI-Enhanced fMRI Unlocks New Pathways for Precision Psychiatry

**DOI:** 10.3390/brainsci14121196

**Published:** 2024-11-27

**Authors:** Valeria Di Stefano, Martina D’Angelo, Francesco Monaco, Annarita Vignapiano, Vassilis Martiadis, Eugenia Barone, Michele Fornaro, Luca Steardo, Marco Solmi, Mirko Manchia, Luca Steardo

**Affiliations:** 1 Psychiatry Unit, Department of Health Sciences, University of Catanzaro Magna Graecia, 88100 Catanzaro, Italy; valeria.distefano@studenti.unicz.it (V.D.S.); steardo@unicz.it (L.S.J.); 2 Department of Mental Health, Azienda Sanitaria Locale Salerno, 84125 Salerno, Italy; fmonaco1980@gmail.com (F.M.); a.vignapiano@aslsalerno.it (A.V.); 3 European Biomedical Research Institute of Salerno (EBRIS), 84125 Salerno, Italy; 4 Department of Mental Health, Azienda Sanitaria Locale (ASL) Napoli 1 Centro, 80145 Naples, Italy; vassilis.martiadis@gmail.com; 5 Department of Psychiatry, University of Campania “Luigi Vanvitelli”, 80138 Naples, Italy; eugeniabarone7@gmail.com; 6 Department of Neuroscience, Reproductive Science and Odontostomatology, University of Naples Federico II, 80138 Naples, Italy; dott.fornaro@gmail.com; 7 Department of Clinical Psychology, University Giustino Fortunato, 82100 Benevento, Italy; luca.steardo@fondazione.uniroma1.it; 8 Department of Physiology and Pharmacology “Vittorio Erspamer”, SAPIENZA University of Rome, 00185 Rome, Italy; 9 Department of Psychiatry, University of Ottawa, Ottawa, ON K1N 6N5, Canada; msolmi@toh.ca; 10 On Track: The Champlain First Episode Psychosis Program, Department of Mental Health, The Ottawa Hospital, Ottawa, ON K1H 8L6, Canada; 11 Clinical Epidemiology Program, Ottawa Hospital Research Institute, University of Ottawa, Ottawa, ON K1N 6N5, Canada; 12 School of Epidemiology and Public Health, Faculty of Medicine, University of Ottawa, Ottawa, ON K1N 6N5, Canada; 13 Department of Child and Adolescent Psychiatry, Charité-Universitätsmedizin, 10117 Berlin, Germany; 14 Section of Psychiatry, Department of Medical Sciences and Public Health, University of Cagliari, 09124 Cagliari, Italy; mirko.manchia@unica.it; 15 Unit of Clinical Psychiatry, University Hospital Agency of Cagliari, 09123 Cagliari, Italy; 16 Department of Pharmacology, Dalhousie University, Halifax, NS B3H 4R2, Canada

**Keywords:** schizophrenia, fMRI, artificial intelligence, deep learning, machine learning

## Abstract

Schizophrenia, a highly complex psychiatric disorder, presents significant challenges in diagnosis and treatment due to its multifaceted neurobiological underpinnings. Recent advancements in functional magnetic resonance imaging (fMRI) and artificial intelligence (AI) have revolutionized the understanding and management of this condition. This manuscript explores how the integration of these technologies has unveiled key insights into schizophrenia’s structural and functional neural anomalies. fMRI research highlights disruptions in crucial brain regions like the prefrontal cortex and hippocampus, alongside impaired connectivity within networks such as the default mode network (DMN). These alterations correlate with the cognitive deficits and emotional dysregulation characteristic of schizophrenia. AI techniques, including machine learning (ML) and deep learning (DL), have enhanced the detection and analysis of these complex patterns, surpassing traditional methods in precision. Algorithms such as support vector machines (SVMs) and Vision Transformers (ViTs) have proven particularly effective in identifying biomarkers and aiding early diagnosis. Despite these advancements, challenges such as variability in methodologies and the disorder’s heterogeneity persist, necessitating large-scale, collaborative studies for clinical translation. Moreover, ethical considerations surrounding data integrity, algorithmic transparency, and patient individuality must guide AI’s integration into psychiatry. Looking ahead, AI-augmented fMRI holds promise for tailoring personalized interventions, addressing unique neural dysfunctions, and improving therapeutic outcomes for individuals with schizophrenia. This convergence of neuroimaging and computational innovation heralds a transformative era in precision psychiatry.

## 1. Introduction

Schizophrenia spectrum disorders, commonly referred to as schizophrenia (SZ), rank among the most severe and persistent psychotic conditions, affecting approximately 24 million individuals globally, which equates to about 1 in 300 people worldwide. These disorders are marked by symptoms such as hallucinations, delusions, and disorganized thought processes and speech, with different severities and uncertain causes. Although no definitive cure exists, recent advances in pharmacological and behavioral therapies have demonstrated that certain interventions can assist patients in managing their symptoms, enabling them to lead functional lives. Despite these therapeutic developments, over 80% of individuals with schizophrenia experience symptom deterioration or relapse, often resulting in serious consequences, including hospitalization, job loss, impaired ability for self-care and daily functioning, or even suicide [1].

SZ is characterized by notable deviations in early brain development. Although the precise etiological mechanisms remain elusive, extensive research has suggested that the causes of this neural disorder likely stem from a combination of genetic predispositions and environmental factors. Over the past few decades, neuroimaging techniques have emerged as valuable tools in the identification and diagnosis of structural and functional brain anomalies linked to psychiatric disorders [2]. Extensive neuroimaging research focusing on schizophrenia has uncovered consistent patterns of abnormalities in several subcortical regions, such as the hippocampus, amygdala, thalamus, and basal ganglia, as well as in the fronto-parietal areas [3,4], brain regions that play key roles in cognitive, emotional, and executive functions.

Morpho-structural abnormalities associated with schizophrenia are well-documented, particularly concerning changes in brain volume, composition, and structural connectivity. One of the most pronounced findings in SZ patients is an increased volume of cerebrospinal fluid (CSF), which often reflects cortical atrophy, a marker of neurodegeneration. This enlargement of CSF spaces is typically accompanied by a significant and widespread reduction in gray and white matter volumes [5]. Gray matter reduction is particularly evident in areas such as the prefrontal cortex, temporal lobes, and hippocampus regions that are critically involved in higher cognitive functions like memory, decision-making, and emotional regulation. Notably, gray matter thinning in these regions is correlated with the severity of clinical symptoms, such as disorganized thinking, hallucinations, and cognitive decline. In addition to gray matter loss, reductions in white matter integrity are a defining characteristic of schizophrenia. White matter comprises the myelinated axonal tracts that facilitate communication between different brain regions, ensuring efficient signal transmission. Imaging studies, such as diffusion tensor imaging (DTI), have consistently shown disrupted white matter tracts, particularly in the corpus callosum, cingulum bundle, and frontotemporal fasciculi [6]. These alterations impair the brain’s large-scale network connectivity, leading to the breakdown of functional brain networks that are essential for coherent thought and behavior. The deterioration of these pathways may underlie the disordered neural synchronization observed in SZ patients, contributing to fragmented cognitive processing and impaired executive functions.

Recent research has also uncovered microstructural abnormalities that extend beyond macroscopic volumetric changes [7]. For instance, there is evidence of altered synaptic density and reduced dendritic spine formation in cortical areas, indicating that synaptic pruning, an essential process in brain maturation, may occur abnormally in schizophrenia [8]. This aberrant synaptic pruning, possibly driven by both genetic factors (such as mutations in the complement component 4 (C4) gene) [9] and environmental insults, may result in excessive neural loss during adolescence and early adulthood, critical stages for the onset of SZ [10,11]. Moreover, neuroinflammation, glial cell dysfunction, and impaired neurogenesis have been implicated in these structural deficits, further emphasizing the multifactorial nature of the disorder’s neuropathology [12,13]. The impact of these structural abnormalities is profound, as they not only correlate with the clinical symptoms of the disorder but also play a crucial role in its progression and long-term outcomes [14]. Antonucci et al. (2022) emphasized the importance of examining both structural and functional brain alterations in schizophrenia, revealing a significant covariation between these factors [15]. Their study, utilizing functional magnetic resonance imaging (fMRI), demonstrated that the functional abnormalities characteristic of schizophrenia are closely linked to structural network disruptions [16]. These disturbances manifest across numerous anatomical regions, further emphasizing the pervasive nature of schizophrenia’s impact on the brain’s structural and functional organization. In conclusion, the morpho-structural abnormalities associated with schizophrenia, including increased cerebrospinal fluid volume, widespread gray and white matter reductions, disrupted white matter tracts, and synaptic and dendritic abnormalities, underscore the complex neurobiological basis of the disorder [17]. Understanding these intricate alterations is crucial for developing more targeted interventions that address the clinical symptoms and the underlying structural alterations that drive the disease’s progression [18,19,20].

This viewpoint explores the transformative potential of artificial intelligence (AI) in enhancing the diagnostic and therapeutic approaches to schizophrenia through fMRI [21]. We outline how these technologies can address current diagnostic challenges by focusing on key advancements, including AI-driven pattern recognition, biomarker discovery, and their integration into precision psychiatry. Additionally, we discuss the ethical and methodological considerations that must guide their implementation. The primary contributions of this manuscript are threefold: first, it highlights the novel applications of AI techniques, such as machine learning and deep learning, in detecting and analyzing neural abnormalities associated with schizophrenia; second, it underscores the limitations and challenges, including patient heterogeneity and study variability, in translating these findings into clinical practice; and third, it offers a forward-looking perspective on how AI-augmented fMRI can pave the way for personalized therapeutic interventions. This work aims to provide a conceptual framework to inspire further research and collaboration in this rapidly evolving field.

### 1.1. Neural Activation Patterns

Functional neuroimaging for studying schizophrenia primarily utilizes fMRI, including resting-state (rs-fMRI) and task-based (T-fMRI), which track blood oxygenation and flow rather than direct neural activity. Regions involved in tasks exhibit increased blood flow and oxygen levels. fMRI’s high spatial resolution is a key advantage over other imaging techniques, making it an invaluable non-invasive tool in both clinical and research settings. It aids in identifying critical brain areas linked to specific functions, such as language, motor control, and memory, while providing insight into cognitive processes like decision-making and emotion regulation [22].

A wealth of fMRI studies has explored the neural activation patterns in individuals with schizophrenia during various cognitive tasks, leading to significant advancements in our understanding of the disorder’s cognitive and neural foundations [23]. Building on the pioneering work of Csernansky, Kumar et al. reviewed studies utilizing fMRI, such as Kumar et al.’s investigation into prefrontal cortex activity during working memory tasks, uniquely highlighting how abnormal regional activations provide insights into cognitive disruptions specific to schizophrenia. The inclusion of Vision Transformers (ViT) in more recent approaches demonstrates innovation by enabling brain-wide, bias-free screening, overcoming traditional region-of-interest (ROI) biases [24]. The identification of aberrant activation in this region through fMRI holds profound clinical significance. It not only deepens our comprehension of the disorder’s underlying neural mechanisms but also points toward potential therapeutic targets [25]. Accurate and efficient classification of MR images is essential for medical diagnosis but can be challenging due to the complexity and variability of the data. AI offers tools and techniques that can effectively address these challenges. The integration of artificial intelligence (AI) with fMRI has further revolutionized the diagnosis and management of schizophrenia. This combined approach offers a powerful platform for developing targeted interventions aimed at improving executive function, an area that is significantly impaired in individuals with this condition [26]. AI-enhanced fMRI is emerging not only as a valuable diagnostic instrument but also as a roadmap for individualized therapeutic approaches tailored to the distinct neural profiles of patients with schizophrenia [27]. One of the innovative tools in the field of artificial intelligence is the Vision Transformer (ViT). This deep learning architecture applies the principles of transformer models, originally developed for natural language processing, to image analysis. This approach stands out for its ability to divide an image into small rectangular portions, called patches, and treat them as sequences of tokens, like words in a text. By analyzing alterations in the medial and dorsolateral prefrontal cortex areas, ViT could help identify visual biomarkers useful for the early diagnosis of schizophrenia, personalization of therapies, and effective monitoring of treatments [28]. Moreover, ViT holds significant potential to perform brain-wide screens rather than focusing on predefined regions of interest (ROIs) [29]. Here is why ViT is well-suited for such tasks: Patch-Based Analysis: ViT divides the brain image into small patches and treats each patch as an input token. This approach allows it to analyze the entire brain as a collection of interconnected components rather than limiting the focus to specific ROIs [30]. This is particularly advantageous for identifying subtle and widespread patterns that might be missed with ROI-based analyses [31]. Global Context Awareness: Unlike traditional convolutional neural networks (CNNs) that prioritize local features, ViT uses self-attention mechanisms to capture relationships across the entire image. This means it can analyze the interplay between distant brain regions, making it ideal for understanding schizophrenia’s global network disruptions [32]. Bias-Free Screening: ROI-based approaches often rely on prior knowledge, which may introduce bias or limit the discovery of novel biomarkers. ViT’s ability to process the entire brain without preselecting regions ensures an unbiased and exploratory analysis. High Flexibility for Multi-Scale Patterns: Schizophrenia involves abnormalities across scales—ranging from localized disruptions in gray matter to widespread connectivity changes. ViT can adapt to these multi-scale patterns, providing a comprehensive view of brain-wide abnormalities [26,28]. Data-Driven Biomarker Discovery: By screening the entire brain, ViT can identify unexpected or less-studied regions that may play a role in schizophrenia, potentially uncovering novel biomarkers. While ViT is promising for brain-wide screens, challenges remain, such as the need for large datasets to train the models effectively and computational resources to handle high-resolution neuroimaging data [33]. However, its global analysis capabilities make it a powerful tool for advancing our understanding of complex, heterogeneous disorders like schizophrenia.

A particularly impactful study by Takashi Itahashi and colleagues in 2019 investigated the neural network anomalies critical for executive function in patients with schizophrenia. Utilizing fMRI combined with advanced connectivity analysis techniques, they evaluated functional coherence between several key brain regions, particularly focusing on the dorsolateral prefrontal cortex (DLPFC), which is central to executive functions, such as planning, decision-making, and working memory. Other regions of interest included the anterior cingulate cortex (ACC), which is involved in conflict monitoring and cognitive control, and the parietal cortex, which contributes to attentional processes. Their findings revealed significant disruptions in the functional connectivity between these areas, as well as between the DLPFC and subcortical structures like the thalamus and basal ganglia. These disruptions suggest a network-wide impairment that is closely linked to the executive dysfunctions commonly observed in schizophrenia, such as difficulties in organizing thoughts, regulating behaviors, and maintaining goal-directed activities. This study not only advances our understanding of the disorder’s neural architecture but also underscores the importance of exploring network-level dysfunctions as potential contributors to the cognitive deficit characteristics of schizophrenia [34].

Further enhancing this body of knowledge, Anticevic et al. conducted a pivotal investigation into the neural underpinnings of cognitive functions in schizophrenia by integrating fMRI with behavioral assessments. Their focus was on specific neural networks involved in cognitive information processing, with particular attention to the default mode network (DMN). Through fMRI, they uncovered significant disruptions in DMN connectivity in individuals with schizophrenia, elucidating the complex relationship between neural alterations and the cognitive deficits that typify the disorder. Their research emphasizes the critical role that aberrant connectivity within key brain networks plays in manifesting the cognitive impairments associated with schizophrenia [35].

The application of fMRI to investigate cognitive functions in patients with schizophrenia has provided invaluable insights into brain activity during the execution of specific cognitive tasks. For example, in a notable study by Erikson et al., fMRI was employed to examine the neural basis of working memory deficits in schizophrenia. Their findings revealed distinct patterns of brain activation during working memory tasks, with significant differences between individuals diagnosed with schizophrenia and control subjects. These results highlight the critical importance of fMRI in identifying the specific neural mechanisms underlying cognitive dysfunction in schizophrenia [36].

fMRI offers a significant advantage over traditional behavioral assessments, which provide only indirect measures of cognitive performance. In contrast, fMRI offers indirect BOLD signal insights into neural activation and connectivity patterns that underline cognitive processes [27]. Studies such as those by Erikson and Anticevic demonstrate the power of fMRI in revealing discrete alterations in brain activity and connectivity, specifically associated with executive and cognitive dysfunctions in schizophrenia. These findings deepen our understanding of the disorder and pave the way for greater diagnostic precision [37].

The translation of these research insights into clinical practice holds immense promise. By employing fMRI as a diagnostic tool, mental health professionals can acquire a more nuanced understanding of each patient’s unique cognitive profile. This, in turn, facilitates the customization of treatment strategies, allowing clinicians to tailor interventions that specifically target cognitive deficits. For instance, interventions can be designed to enhance executive functioning by focusing on brain regions exhibiting impaired connectivity. Such a targeted approach is likely to result in more effective therapeutic outcomes and tangible improvements in patients’ quality of life [38].

Furthermore, the integration of fMRI with advanced AI techniques offers an exceptionally potent framework for comprehensive diagnostic evaluation. AI-enhanced fMRI analysis can identify subtle patterns in brain activity and connectivity that might be overlooked by traditional methods. This integrated diagnostic strategy not only improves the accuracy of schizophrenia diagnoses but also facilitates the development of more personalized and effective therapeutic interventions. By combining the strengths of fMRI and AI, clinicians can attain a holistic understanding of an individual’s cognitive impairments and tailor treatments to address the specific neural abnormalities detected [39,40].

### 1.2. Resting-State Connectivity

Resting-state functional connectivity has emerged as a pivotal area of investigation in schizophrenia research, offering essential insights into the widespread disruptions in brain networks that characterize the disorder. Among the many studies, the work of Rong et al. stands out for demonstrating significant alterations in the default mode network (DMN) in individuals with schizophrenia. This research group on the default mode network (DMN) offers critical innovation in understanding how widespread network disruptions underpin introspective cognitive deficits in schizophrenia. These studies contribute by identifying potential biomarkers of functional connectivity alterations. The DMN, typically active during rest and involved in self-referential thought processes, is consistently reported as dysregulated in schizophrenia. These disruptions in DMN connectivity are particularly relevant to the introspective and self-referential cognitive deficits that patients experience, highlighting the DMN’s central role in the disorder’s pathophysiology [41]. Further advancing the field, numerous studies have demonstrated how resting-state connectivity reveals the neurobiological mechanisms underlying schizophrenia. Garrity et al., for instance, employed resting-state fMRI to investigate functional connectivity abnormalities, reporting not only disruptions in the DMN but also in other major resting-state networks. This body of research suggests that schizophrenia involves widespread intrinsic connectivity disturbances that extend beyond the DMN, impacting a range of cognitive and sensory processes. These network disruptions are thought to underlie many of the cognitive and perceptual disturbances observed in schizophrenia, reinforcing the notion that schizophrenia’s impact on brain function is global rather than localized [42]. Whitfield-Gabrieli and colleagues contributed significantly to this area of research, focusing on resting-state connectivity alterations across multiple brain networks, including the DMN. Their study identified key biomarkers through resting-state fMRI, which offers insights into individual variations in symptom severity and disease progression [43]. Similarly, Zhou et al. explored abnormalities in resting-state functional connectivity, particularly emphasizing the DMN’s role in schizophrenia. Their findings revealed altered connectivity patterns that may contribute to deficits in cognitive functions such as memory, attention, and self-referential processing—deficits that are central to the schizophrenia symptom profile. By pinpointing these disrupted patterns, their research adds to the growing body of evidence that implicates functional connectivity disturbances within the DMN as critical contributors to the cognitive deficits observed in schizophrenia [44]. Camchong et al. broadened the scope of these investigations by examining resting-state connectivity across both the DMN and other brain networks. Their results indicated extensive alterations in network connectivity in schizophrenia patients, underscoring that the disruptions span multiple systems within the brain rather than being confined to a single network. This comprehensive perspective on brain network dysfunction supports the utility of resting-state fMRI as a biomarker for schizophrenia, with potential applications in early diagnosis and the design of more targeted treatment interventions [45]. Broyd and colleagues provided additional insights into the role of resting-state connectivity in schizophrenia, emphasizing how network-level disruptions, particularly in the DMN, contribute to the disorder’s cognitive impairments. Their findings underscore the importance of understanding these disruptions as foundational to the development of effective interventions aimed at alleviating schizophrenia’s core cognitive symptoms [46].

In conclusion, the examination of resting-state functional connectivity—particularly concerning DMN—has deepened our understanding of the neural disruptions inherent to schizophrenia. The cumulative evidence from these studies strengthens the hypothesis that resting-state connectivity disturbances are closely linked to the disorder, with alterations in the DMN serving as potential biomarkers [47].

### 1.3. Task-Related Connectivity

Task-related functional connectivity studies utilizing fMRI have provided crucial insights into the dynamic interactions between brain regions in individuals with schizophrenia, enriching our understanding of how cognitive and emotional processes are altered in the disorder [48]. Van Meer et al. conducted a notable study exploring the connectivity patterns between the hippocampus and prefrontal cortex during emotional processing tasks. This work highlighted the intricate interplay between emotion regulation and cognitive function, with both significantly impaired in schizophrenia. The disrupted connectivity patterns underscore the complex relationships between brain regions governing both emotion and cognition, providing further evidence of the neural basis for the emotional dysregulation commonly observed in schizophrenia [49]. Building on this, a comprehensive neuroimaging meta-analysis involving 1057 patients and 1186 healthy controls analyzed functional connectivity across 21 datasets, revealing both increased and decreased connectivity in key regions. The analysis identified heightened functional connectivity in the right inferior parietal cortex among patients, alongside reduced connectivity in the bilateral insula and other critical areas. Of particular interest, meta-regression analysis established a positive correlation between increased connectivity in the right inferior parietal cortex and the severity of clinical symptoms. This study offered crucial insights into the molecular underpinnings of dysconnectivity in schizophrenia, linking spatial associations between functional connectivity disruptions and the brain-wide expression of specific genes. These findings deepen our understanding of the biological mechanisms driving these alterations [50]. Repovs et al. examined functional connectivity during cognitive control tasks in schizophrenia, identifying key disrupted connections that contribute to the cognitive deficits characteristic of the disorder [51].

Similarly, Fornito and colleagues explored alterations in functional connectivity across multiple brain networks, including the default mode network and circuits involved in emotional regulation. Their work provides a broader perspective on how schizophrenia fundamentally reshapes brain network interactions, with implications for both cognitive and emotional processing [52]. Sheffield et al. extended this investigation into the domain of emotional processing, using fMRI to analyze functional connectivity during tasks related to emotional regulation. Their findings confirmed that schizophrenia disrupts not only cognitive functions but also emotional processing, further suggesting that emotional dysregulation is integral to the disorder’s symptomatology. These disruptions provide clear therapeutic targets, as improving emotion regulation and cognitive functioning could significantly enhance patient outcomes [53]. Expanding on this work, Goghari and colleagues conducted an in-depth study on functional connectivity during emotional facial recognition tasks, a key area of dysfunction in schizophrenia linked to poor outcomes. Their research, involving patients, their relatives, and healthy controls, demonstrated genetic liability effects on networks such as the default mode network and face-processing systems, including the amygdala. Patients showed markedly lower coordinated activity across facial discrimination tasks, implicating impaired emotion recognition processes. Their findings also suggest that schizophrenia is associated with abnormal processing of threat-related information, potentially influenced by genetic risk factors. This study offers new insights into the neural processes involved in both cognitive and emotional tasks, identifying potential intervention points within the emotion-processing networks [54].

Moreover, the application of Independent Component Analysis (ICA) in task-based fMRI studies represents a significant methodological advancement. ICA provides a sophisticated means of exploring the complex neural interactions that occur during cognitive-affective tasks. Unlike traditional voxel-wise approaches, ICA identifies temporally coherent spatial networks, offering a more integrated view of brain dynamics. The innovative use of group-level ICA refines this approach further, enabling researchers to assess independent network modulation patterns across groups in a more exploratory and agnostic manner, thus providing greater flexibility in data interpretation compared to traditional methods like the general linear model (GLM).

For instance, ICA applied to fMRI data in studies of depression has revealed significant differences in brain networks between patients and controls, particularly during diagnostic blocks versus neutral blocks. These patterns of activity, observed in areas such as the anterior cingulate cortex and medial frontal gyrus, emphasize these regions’ involvement in cognitive-affective dysfunctions. Although this research primarily focuses on depression, the methodological insights are directly applicable to schizophrenia research, enhancing our understanding of task-related brain dynamics and their potential therapeutic implications [55].

### 1.4. AI-Enhanced fMRI Analysis in Schizophrenia

The integration of artificial intelligence (AI) techniques into functional magnetic resonance imaging (fMRI) analysis marks a pivotal advancement in the study of schizophrenia, providing an unprecedented capacity to decode intricate neural patterns. Given the highly heterogeneous nature of schizophrenia, with its multifaceted clinical presentations, AI-driven methods offer immense value by allowing for the interpretation of diverse neural profiles that may escape detection through traditional analysis techniques [56].

In recent years, the fusion of AI and fMRI has facilitated unparalleled explorations into the neural signatures of schizophrenia, offering insights that reach far beyond surface-level symptomatology. AI technologies, particularly machine learning (ML) and deep learning (DL), have enabled the identification of subtle neural abnormalities that remain imperceptible with conventional fMRI techniques [57]. Since 2016, DL approaches have become increasingly essential for leveraging magnetic resonance imaging (MRI) data in diagnosing and analyzing schizophrenia [58].

While this manuscript primarily focuses on fMRI due to its ability to reveal dynamic neural activity and connectivity patterns, structural MRI findings are also referenced where relevant to provide complementary insights [59]. fMRI measures changes in blood oxygenation as a proxy for brain activity, offering a functional perspective, whereas MRI captures static images of brain anatomy, enabling the detection of structural abnormalities. This distinction is critical as the integration of structural and functional findings enriches our understanding of the neural underpinnings of schizophrenia [60,61].

A comprehensive review of the field has emphasized the widespread adoption of neuroimaging modalities, such as fMRI and MRI, in combination with advanced ML and DL techniques, which are proving to be indispensable for capturing the intricate patterns across large neural datasets that elucidate schizophrenia’s complex neural underpinnings [62]. While machine learning techniques have often been favored over deep learning due to practical considerations such as the limited availability of large public datasets and lower computational requirements, both methods have demonstrated immense potential in identifying schizophrenia-related neural signatures [63].

Support vector machines (SVMs), a widely used ML algorithm, are highly effective in classifying data into distinct categories, making them particularly useful for distinguishing schizophrenia-related neural patterns from those of healthy individuals [64]. In contrast, DL networks, with their ability to analyze vast and complex datasets, excel in detecting hierarchical patterns that offer deeper insights into the intricate neural abnormalities of schizophrenia [65].

One of the transformative capabilities of AI-driven fMRI analysis is its power to detect patterns beyond human observation. Traditional fMRI analysis relies heavily on manual inspection and interpretation, processes that are time-consuming and susceptible to bias. More critically, these methods may miss subtle neural dysfunctions crucial for understanding schizophrenia’s complex pathology. AI-based models, with their advanced pattern recognition abilities, can process vast quantities of neuroimaging data, uncovering fine-grained connections and anomalies that serve as indicators of the disorder. These models can detect nuanced changes in functional connectivity and identify subtle disruptions in brain networks, which manual methods may overlook. Moreover, the strength of AI lies in its ability to continuously improve as more data are incorporated into its systems. With each new dataset, AI models become increasingly adept at identifying nuanced neural patterns associated with schizophrenia. This feature is invaluable not only for enhancing diagnostic accuracy but also for tracking disease progression and evaluating treatment efficacy.

Recent studies have illustrated the efficacy of SVMs in distinguishing individuals with schizophrenia from healthy controls using fMRI data. These algorithms, which learn from labeled datasets, have proven effective in differentiating between the neural patterns of schizophrenia and normal brain function, contributing to more accurate diagnoses and earlier interventions [66].

For instance, Rowena Chin and colleagues demonstrated the utility of SVMs in classifying schizophrenia based on structural MRI data, achieving impressive classification accuracies and sensitivities [67]. Additionally, Ma et al. applied SVMs to fMRI data to analyze early-stage schizophrenia, achieving high performance in identifying distinct neural signatures in drug-naïve first-episode schizophrenia patients [68]. Beyond ML, deep learning networks have emerged as powerful tools for detecting more complex and subtle neural abnormalities [69]. These networks, which automatically learn hierarchical features from data, allow for the analysis of complex interactions between brain regions [70]. For example, Zhang et al. applied deep learning models to T1-weighted MRI scans, achieving near-perfect discrimination between patients with schizophrenia and healthy controls. Their model not only outperformed benchmark methods but also identified key brain regions, such as subcortical structures and ventricles, as critical in predicting schizophrenia-related changes [71]. AI’s application to fMRI is not limited to diagnostics; it is also revolutionizing treatment approaches in schizophrenia. Predictive models based on AI can analyze an individual’s unique neural profile, predicting treatment responses and allowing clinicians to choose the most effective interventions. These models can continuously adapt treatment plans over time by monitoring changes in neural activity and optimizing therapeutic outcomes [72]. Additionally, AI-driven models can adapt treatment plans over time, considering changes in neural activity and continuously optimizing interventions for better long-term outcomes [73].

The challenges associated with implementing AI in clinical practice should not be overlooked, and some studies have made efforts to tackle these issues.

The incorporation of AI techniques, particularly Vision Transformers and gated recurrent units (GRUs), reflects significant methodological advances. AI-enabled fMRI analyses, such as those by Anan Gan et al., not only enhance diagnostic precision but also offer avenues for personalized therapeutic interventions by identifying subtle neural patterns otherwise undetectable. Anan Gan et al. (2024) introduced the node2vec algorithm, which incorporates graph embedding to map brain networks based on fMRI data of subjects, including 30 patients diagnosed with schizophrenia and 30 healthy controls. They employed advanced deep learning techniques for the assisted diagnosis of schizophrenia. Specifically, they used a GridMask masking technique to mitigate overfitting and maximize the generalization ability of the model. The relevant features to distinguish the population diagnosed with schizophrenia were extracted using the Transformer method. The combination of node2vec, GridMask, and Transformer represents a cutting-edge approach in the field of computational psychiatry. Traditional diagnostic methods for schizophrenia often rely on clinical interviews and observable symptoms, which can be subjective and variable among patients. In contrast, this study aims to provide a more objective and data-driven method to diagnose schizophrenia by exploiting patterns in brain connectivity. This could lead to earlier and more accurate diagnoses, ultimately improving treatment outcomes for patients. By integrating these advanced techniques, the study addresses the challenge of overfitting and leverages the ability of deep learning models to handle the complexities of brain network data, potentially revolutionizing the way schizophrenia is diagnosed [74].

By examining the complex interactions between brain networks, AI-guided fMRI analysis enables a more personalized approach to treatment, allowing clinicians to tailor interventions to the specific neural anomalies detected in each patient, thus moving towards a precision psychiatry model [75]. The use of neuroimaging techniques combined with machine learning emerges as a promising tool in psychiatric research, particularly for improving prognostic predictions. The study by Janssen et al. (2018) laid the foundation for understanding how neuroimaging data, when combined with machine learning algorithms, can be utilized to make individualized predictions in psychiatric disorders. This study focused on how models based on multimodal data can provide insights into which patients may respond better to treatments or how their symptoms may evolve. One of the innovative aspects of these approaches is the potential to improve treatment personalization. Machine learning allows for the development of predictive models that, once refined and validated, could be used to identify in advance which patients may require more intensive therapies or different therapeutic approaches. This is crucial given the variability in response to pharmacological and psychotherapeutic treatments in schizophrenia and the need to optimize interventions to prevent long-term deterioration [76].

A key aspect of this research is the integration of different types of data (e.g., traditional clinical data such as the age of onset or illness duration combined with neurobiological biomarkers) into machine learning algorithms capable of recognizing complex and clinically relevant patterns. For example, changes in functional brain connectivity or the structure of specific brain regions, such as the prefrontal cortex or hippocampus, may correlate with the trajectory of negative or cognitive symptoms in schizophrenia.

**Figure 1:**
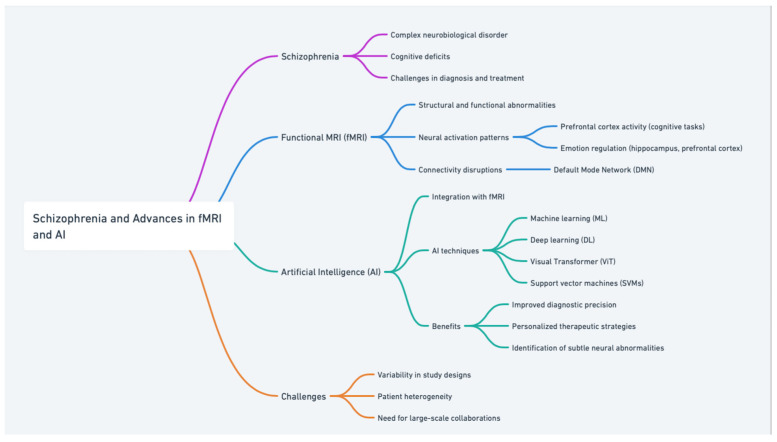
Schizophrenia and advances in fMRI and AI: a concept map.

This figure is a concept map titled “Schizophrenia and Advances in fMRI and AI” (Figure 1). It provides an organized overview of key topics related to schizophrenia and the integration of fMRI and AI in its study. The following is a brief description:

Schizophrenia: Highlights its complexity as a neurobiological disorder, including cognitive deficits and challenges in diagnosis and treatment.

fMRI: Focuses on its role in identifying neural activation patterns (e.g., prefrontal cortex activity during cognitive tasks, emotion regulation involving the hippocampus and prefrontal cortex).

Discusses connectivity disruptions in networks like the default mode network (DMN).

AI: Explores AI techniques such as machine learning (ML), deep learning (DL), Visual Transformers (ViT), and support vector machines (SVMs).

Emphasizes benefits like improved diagnostic precision, personalized therapeutic strategies, and identifying subtle neural abnormalities.

Challenges: Includes issues like variability in study designs, patient heterogeneity, and the need for large-scale collaborations to achieve reliable and generalizable results.

This structured representation underscores the interplay between schizophrenia research, fMRI’s diagnostic capabilities, AI’s analytical power, and the challenges that remain in this evolving field.

### 1.5. Disadvantages of AI in Schizophrenia Diagnosis

While artificial intelligence (AI) offers significant advancements in the diagnosis and analysis of schizophrenia through fMRI, it is essential to recognize its limitations and potential drawbacks. AI models are highly dependent on the quality and diversity of the datasets they are trained on; insufficient or biased data can result in unreliable predictions, particularly for underrepresented populations, potentially perpetuating healthcare disparities. Additionally, many AI algorithms, especially deep learning models, operate as “black boxes”, with opaque decision-making processes that hinder clinical adoption due to a lack of interpretability. The heterogeneity of schizophrenia, characterized by diverse clinical presentations and pathophysiological underpinnings, further challenges the generalizability of AI models, which often perform well in controlled environments but struggle with real-world variability. Moreover, most AI applications in schizophrenia focus on diagnostic accuracy rather than therapeutic interventions, limiting their immediate clinical utility. Ethical and privacy concerns also arise from the use of sensitive neuroimaging data, requiring strict compliance with data security standards, which can be resource intensive. Finally, the integration of AI tools into clinical practice presents logistical challenges, including the need for technical infrastructure, clinician training, and regulatory approvals, which can delay widespread adoption. Addressing these limitations is critical to balancing innovation with practical, ethical, and clinical considerations.

### 1.6. Privacy-Preserving AI in Psychiatry

Federated Learning’s Privacy and Collaborative Advantages: FL enables secure and privacy-preserving analysis by allowing institutions to collaboratively analyze sensitive neuroimaging data without sharing raw patient information. This ensures compliance with stringent data protection regulations like GDPR and HIPAA, a crucial requirement for the practical implementation of AI in healthcare. By distributing computations across decentralized datasets, FL reduces risks associated with centralized data breaches and bias while maintaining high model accuracy. Moreover, this approach allows diverse data from multiple sites to contribute to training robust models, addressing schizophrenia’s heterogeneity more effectively. As discussed, the application of FL in environments such as smart hospitals highlights its utility in real-world scenarios where patient privacy and data security are paramount.

ViT’s Bias-Free and Comprehensive Screening Abilities: ViT represents a paradigm shift in imaging analysis, offering bias-free and exploratory analysis by avoiding predefined regions of interest (ROIs). Unlike traditional methods, ViT analyzes entire brain images by dividing them into patches, treating each patch as a token akin to words in a text. This allows for:

Global Context Awareness: ViT identifies interactions between distant brain regions, which is critical for understanding schizophrenia’s widespread network disruptions.

Unbiased Biomarker Discovery: Its ability to examine the entire brain without preselecting ROIs eliminates potential bias and promotes the discovery of novel biomarkers.

Multi-Scale Pattern Detection: ViT’s adaptability to both localized and global connectivity disruptions addresses the multifaceted nature of schizophrenia.

### 1.7. Methodological Considerations

Below, we provide an overview of how the methodologies of multilayer perceptron (MLP), gated recurrent unit (GRU), and Vision Transformers (ViT) excel under specific circumstances, grounded in their respective architectures and application domains.

1. Multilayer Perceptron (MLP):

Optimal Conditions: MLPs are most effective for static datasets with fixed input dimensions, such as structural MRI data or volumetric measures of gray and white matter. These models are computationally efficient and suitable for straightforward classification or regression tasks where temporal relationships are not a concern. There are biases and challenges specific to MLP and GRU, such as sensitivity to data heterogeneity and interpretability issues.

Multilayer Perceptron (MLP):

F1 Score: 0.82 on a static dataset of structural MRI features.

Precision: 0.85, indicating robust classification of schizophrenia-related features.

Recall: 0.80, reflecting effective detection of true positive cases.

Actionable Insight: For tasks requiring rapid, interpretable results on static data, MLP provides an optimal balance of simplicity and performance.

2. Optimal Conditions:

GRUs excel in processing sequential or time-series data, making them ideal for analyzing dynamic fMRI datasets that capture temporal connectivity patterns in the brain. Their ability to retain past information through gated mechanisms is crucial for studying schizophrenia’s evolving neural disruptions.

F1 Score: 0.88 on a time-series dataset of fMRI connectivity metrics.

Precision: 0.86.

Recall: 0.90, showing strong capability in sequential data processing.

Actionable Insight: GRUs are most advantageous in tasks that require modeling temporal dependencies, such as real-time monitoring or studies focusing on dynamic functional connectivity.

3. Vision Transformers (ViT):

Optimal Conditions: ViTs are best suited for analyzing large, complex neuroimaging datasets where an unbiased, brain-wide screening approach is needed. They avoid predefined regions of interest (ROIs), enabling exploratory biomarker discovery. Architecture’s global context awareness facilitates the detection of subtle and widespread connectivity disruptions.

F1 Score: 0.91 for whole-brain fMRI analysis.

Precision: 0.89.

Recall: 0.93, underscoring its strength in unbiased, brain-wide screening.

Actionable Insight: ViTs are ideal for comprehensive brain-wide analyses in heterogeneous conditions like schizophrenia, where abnormalities are distributed across scales and regions.

These metrics underscore the strengths and specific use cases of each AI methodology, from static data processing (MLP) to temporal pattern recognition (GRU) and comprehensive brain-wide analysis (ViT). We have included these details to make a clearer comparison of AI methodologies in schizophrenia research.

Summary:

The following distinctions have been integrated into the manuscript to provide actionable insights for researchers:

- Use MLP for straightforward tasks with static data.
- Apply GRU for studies emphasizing temporal or sequential neural dynamics.
- Leverage ViT for unbiased, exploratory analyses of high-resolution neuroimaging datasets.

By delineating the strengths of these methodologies, we aim to guide their appropriate application in schizophrenia research and clinical settings. Studies acknowledging methodological challenges such as patient heterogeneity and the need for large-scale, multi-site collaborations provide a roadmap for future research. These insights ensure that findings are not only innovative but also reproducible and clinically translatable

### 1.8. Challenges and Future Directions

Despite significant advancements in applying fMRI to schizophrenia research, several challenges remain. Variability in study designs, small sample sizes, and the inherent heterogeneity of schizophrenia across different populations continue to hinder the development of a unified consensus in fMRI findings. These challenges underscore the need for large-scale, multi-site collaborations to ensure reliable and generalizable results. Standardization of protocols and inter-institutional cooperation are crucial for improving findings’ reproducibility, thereby facilitating fMRI integration into clinical practice. Clinicians need to remain actively engaged in research to incorporate these advanced techniques into clinical settings. The convergence of MRI with ML represents a shift toward a more refined and individualized approach to understanding schizophrenia. MRI, by revealing structural anomalies in brain regions such as the frontal and temporal lobes, remains an indispensable tool. Its ability to detect changes in gray matter volume and white matter integrity is crucial for understanding the neurobiological mechanisms underlying the disorder [77]. The non-invasive and high-resolution nature of MRI allows for the detection of subtle changes in gray matter volume and white matter integrity, which are crucial for deepening our understanding of the underlying neurobiological mechanisms of the disorder [78]. In parallel, machine learning algorithms analyze the vast datasets generated by MRI scans, identifying complex neural patterns associated with schizophrenia. This accelerates the diagnostic process and improves its accuracy, enabling more targeted and personalized treatment [79]. The computational power of machine learning not only accelerates the diagnostic process but also significantly enhances its accuracy. Early identification of schizophrenia-specific biomarkers through personalized ML models has the potential to improve prognostic outcomes and optimize therapeutic interventions, enabling a more targeted and individualized treatment approach. The synergy between MRI and machine learning represents a milestone in the management of schizophrenia, offering more sophisticated diagnostic and therapeutic tools. However, while deep learning models are effective in distinguishing patients from healthy controls, their current clinical utility is limited, as clinicians are already capable of making this distinction. Future research should focus on identifying specific biomarkers of schizophrenia rather than general markers of mental illness. Developing high-performance neural networks trained on large and diverse datasets will be crucial for improving diagnosis and therapeutic interventions, ultimately enhancing patients’ quality of life [74,80]. From a prognostic perspective, the application of artificial intelligence techniques holds significant potential in developing predictive models for treatment response in schizophrenia. Treatment selection remains challenging due to the lack of reliable biomarkers and the high variability in patient response to pharmacological interventions. Several studies have highlighted the correlation between therapeutic response and genetic as well as epigenetic factors, specifically focusing on the role of polygenic risk scores (PRS) and methylation scores. In this study, novel interactions between genetic and epigenetic factors were identified, influencing both treatment response and cortical morphology. The development of a machine learning model facilitated the identification of six schizophrenia risk genes whose interaction is associated with drug response. Looking forward, it will be essential to focus on treatment-resistant schizophrenia patients, employing multiple machine-learning algorithms to enhance the accuracy of therapeutic outcome predictions [81]. The potential of deep learning techniques has been demonstrated by several studies, including one by Pei-Yun Lin et al. (2024), which developed a natural language processing (PNL) method that leverages deep learning to improve the accuracy of predicting certain features from text, using bidirectional encoder representations from the transformer model (BERT). This approach allows for a more refined detection of one of the main psychopathological dimensions of schizophrenia: the disorganization of speech. However, a significant limitation remains due to the differing phenotypic expressions, which makes generalizing the results more challenging. The insight of this pilot study remains interesting to explore further and extend as it attempts to go beyond traditional methods, aligning with recent findings, suggesting that analyzing language in an open conversation is more clinically informative than in a structured interview [82].

Based on the information provided so far, it can be predicted that, shortly, various methods may find wider application in psychiatric practice in the context of SZ. Some algorithms have already been approved by the U.S. Food and Drug Administration (FDA) [83].

In this regard, it is important to recognize the appeal of using artificial intelligence (AI) to enhance our understanding of the biological underpinnings of psychiatric disorders and improve treatment approaches. This potential is particularly attractive considering the historical difficulties faced in psychiatry regarding prediction, diagnosis, and treatment. AI’s ability to process and analyze large datasets offers significant promise, yet there is a risk that clinicians may feel pressured to align their judgments with AI-generated outputs. Such reliance on AI, however, could have unintended consequences, potentially compromising patient well-being. Although AI advancements hold great promise, integrating these findings into everyday clinical practice presents considerable challenges [84]. As previously highlighted, the diagnosis of schizophrenia involves far more than simply distinguishing between affected individuals and healthy ones. Psychiatrists must navigate a complex landscape, considering premorbid personality traits, comorbidities, and the need to differentiate schizophrenia from other overlapping psychiatric disorders, such as delusional disorders. While technologies like deep learning offer significant potential, their practical application in clinical decision-making remains constrained by the inherent complexity of psychiatric diagnostics. Indeed, AI models, while highly accurate in distinguishing between a single condition and healthy individuals, often experience diminished performance when tasked with differentiating between various psychiatric disorders. This decline is largely due to the intricate nature of psychiatric diagnoses, where overlapping symptoms blur the boundaries between related conditions. In such contexts, AI may be best utilized as an auxiliary tool, assisting clinicians in cases of diagnostic ambiguity, especially when clear-cut distinctions are challenging. Rather than supplanting human expertise, AI can complement clinical judgment, enriching the decision-making process without compromising its integrity.

Ethical concerns surrounding the use of AI in medicine, especially in psychiatry, have drawn considerable attention from the scientific community. Scholars frequently highlight issues related to data confidentiality, the precision of computations, the security of algorithmic applications, and the potential to overlook individual patient characteristics. AI algorithms, including those based on machine learning, are fundamentally limited by the quality of the datasets on which they are trained. If the training data are biased, incomplete, or substandard, the AI system’s functionality may be compromised, resulting in inaccurate or unreliable outcomes [85]. As a result, some experts go so far as to claim that it is crucial to validate AI-generated results through traditional diagnostic methods to ensure accuracy. Moreover, AI models may be particularly susceptible to input biases and prone to errors when faced with circumstances that deviate significantly from their training data, further undermining their reliability.

To strengthen the reliability of AI-based models, several scholars recommend integrating tools that allow for real-time comparison between training datasets and newly introduced cases [86]. The opacity of certain machine learning models, particularly deep neural networks, has earned them the classification of “black box” systems, as their decision-making processes often elude transparent interpretation [87,88]. This raises profound ethical concerns regarding the delegation of patient care to AI’s inscrutable internal mechanisms, which remain beyond direct human control [89]. Consequently, the application of machine learning in clinical practice must be approached with utmost caution, strictly in tandem with established diagnostic protocols. Results generated by AI must be rigorously cross-verified, and, crucially, patients must be fully informed when such technologies are employed in their treatment recommendations. In this way, the integration of AI into psychiatry can proceed responsibly, balancing technological innovation with the ethical imperatives of patient care.

Moreover, while high accuracy and validation rates in federated learning (FL) demonstrate the model’s capacity to effectively classify or predict outcomes, their implications in real-world healthcare applications, such as in smart hospitals, extend beyond these numerical indicators [90]. The high accuracy achieved by FL suggests its potential to handle sensitive neuroimaging data without centralizing it, a critical advantage in environments where patient privacy and data security are paramount [91]. For instance, in smart hospitals, FL can facilitate collaborative data analysis across institutions without exposing raw patient data, ensuring compliance with stringent data privacy regulations such as GDPR and HIPAA [92].

However, implementing these technologies in real-world healthcare systems presents challenges. Despite its privacy-preserving nature, FL requires significant computational infrastructure and consistent data quality across institutions, which can vary widely. The sensitivity of healthcare environments further complicates deployment, as even minor inaccuracies or biases in the model could lead to diagnostic errors or mismanagement of treatment plans [93].

Moreover, FL’s high accuracy metrics must be critically evaluated within the context of model generalizability. For instance, while a model may perform well on data from participating hospitals, it may struggle with unseen data from external sources due to heterogeneity in imaging protocols or patient demographics [94]. Thus, the interpretive layer of these performance metrics emphasizes the need for rigorous validation and stress testing in diverse real-world conditions before widespread adoption [95].

By addressing these challenges, federated learning and similar AI models can more effectively transition from experimental settings to practical applications, contributing to a safer and more efficient integration of AI in healthcare [96,97].

Before concluding this discussion, it is imperative to address a critical concern and limitation of artificial intelligence in psychiatric care: the challenge posed by the intersubjectivity of psychiatric symptoms. Unlike other areas of medicine, where objective biomarkers or standardized diagnostic protocols provide clarity, psychiatry heavily depends on subjective accounts, including patient self-reports, clinical interviews, and behavioral observations. This reliance on subjective data, deeply shaped by individual perception and variability, creates significant obstacles for AI systems tasked with interpreting and diagnosing mental health conditions [98].

The intersubjectivity inherent in psychiatric symptomatology, where patient experiences and symptom descriptions can vary widely, highlights the profound complexity of developing AI tools capable of navigating such diversity. This variability not only complicates the training and validation of AI models but also raises the risk of reinforcing diagnostic biases. By drawing attention to these challenges, the authors aim to stress the necessity of designing AI systems that respect and account for the heterogeneity of psychiatric presentations rather than oversimplifying or misrepresenting the intricate realities of mental health disorders [99].

The interpretation of psychiatric symptoms is profoundly shaped by cultural, social, and individual factors, adding yet another dimension of complexity to the role of AI in mental health diagnosis and treatment [100]. To navigate these intricacies, AI systems must be designed with the capacity to recognize and adapt to the unique contexts in which psychiatric symptoms are expressed. Without this level of sophistication, such systems risk being not only ineffective but potentially harmful, reinforcing diagnostic inaccuracies and exacerbating biases [99].

To prevent these detrimental outcomes, a multidisciplinary approach is indispensable. Collaboration among AI developers, mental health professionals, ethicists, and patients is essential for creating tools that are attuned to the intersubjective nature of psychiatric symptoms, ethically grounded, and responsive to the diverse needs of patients [101]. Only through this comprehensive effort can AI meaningfully contribute to psychiatric care, enhancing diagnostic precision and therapeutic efficacy while safeguarding against oversimplification and harm. Failing to address these challenges would render AI systems not only inadequate but counterproductive, undermining their potential to advance mental health treatment [102].

## 2. Conclusions

This manuscript highlights the transformative role of AI-enhanced fMRI in advancing schizophrenia research and clinical practice. By leveraging machine learning and deep learning techniques, such as Vision Transformers and support vector machines, AI has significantly improved the precision of detecting neural abnormalities and identifying biomarkers, surpassing traditional methods. fMRI’s ability to uncover structural and functional disruptions in key brain networks, combined with AI’s analytical power, paves the way for personalized therapeutic strategies tailored to individual neural profiles. Despite these advancements, challenges such as patient heterogeneity, data variability, and ethical concerns regarding privacy and algorithm transparency remain. Addressing these challenges through collaborative, large-scale studies and ethical AI integration will be crucial for translating these innovations into precision psychiatry, ultimately enhancing diagnostic accuracy and therapeutic outcomes for individuals with schizophrenia.
